# Epidemiology of Periodontal Disease

**DOI:** 10.1155/2012/848641

**Published:** 2012-12-16

**Authors:** Fernando Oliveira Costa, Cristiano Susin, Jose Roberto Cortelli, Isabela Almeida Pordeus

**Affiliations:** ^1^Federal University of Minas Gerais, Contorno Avenue 4849, 30110031 Belo Horizonte, MG, Brazil; ^2^Laboratory for Applied Periodontal & Craniofacial Regeneration, Center for Clinical and Translational Craniofacial Research, College of Dental Medicine, Georgia Health Sciences University, USA; ^3^University of Taubaté, Taubaté, SP, Brazil

Epidemiology is the study of health and disease in populations and of how these states are influenced by biology, heredity, and physical and social environment, as well as personal behavior. Advances in research over recent years have led to a fundamental change in our understanding of the periodontal diseases. As recently as the mid-1960s, the prevailing model for the epidemiology of periodontal diseases included the following precepts: (1) all individuals were considered more or less equally susceptible to severe periodontitis; (2) gingivitis usually progressed to periodontitis with consequent loss of bone support and eventually loss of teeth; (3) susceptibility to periodontitis increased with age and was the main cause of tooth loss after age 35–55. Since the development of this paradigm, advances in the understanding of periodontal diseases have led this disease model to be reevaluated. Current knowledge has shown that periodontitis does not present a linear progression and is not age dependent. Moreover, its distribution and severity are strongly influenced by host susceptibility and risk factors. Several epidemiological studies evaluating destructive periodontal diseases have been pursuing associations in the incessant identification of risk factors for these diseases. Analytical epidemiology seeks to identify the risk factors associated with a disease, to quantify the strength of those associations and to estimate whether an association is causal. An understanding of risk factors can lead to theories of causation and then to treatment protocols for clinicians to use in their daily practice. The essential features of epidemiology as a method of research, when compared to clinical research and case studies, are that (1) groups rather than individuals are the focus of study; (2) persons with and without a particular disease (e.g., periodontal diseases) and with and without the exposure of interest are included, rather than just patients. The study of population groups rather than individuals is to allow for valid estimates while accounting for normal biological variation (e.g., some individuals form dental biofilm readily; others do not). Broadening a study to include those with and without a disease can provide a reference point against which a risk is quantified.

Under such background, this special issue was designed to summarize the current status of periodontal epidemiology. Thus, we have invited authors to submit original research articles and analysis that seek to clarify aspects of the epidemiology of periodontal diseases and the relevant related factors. As a result, various interesting and scientifically significant papers have been accepted. This special supplement presents manuscripts reporting information about the prevalence and distribution of periodontitis, as well as their associations with risk factors. In addition several studies reported tooth loss, one of the most visible results of the evolution of periodontitis that causes physiological and psychological impacts on patient's life.

These papers represent an exciting and insightful observation into the state of art, as well as emerging future topics, in this important interdisciplinary field. We hope that this special issue would attract a major attention of the peers. 

